# Shenlian (SL) Decoction Treat Diabetic Nephropathy by Regulating M1 Polarization of Macrophages and Pyroptosis of Renal Tubular Epithelial Cells

**DOI:** 10.1155/jdr/1757275

**Published:** 2026-04-27

**Authors:** Xiaozhe Fu, Qiang Fu, Yuanyuan Liu, Yifan Liu, Junheng Wang, Jie Hu, Chuxiao Tian, Wenqian Yu, Yixuan Ren, Weijun Huang, Jinxi Zhao

**Affiliations:** ^1^ Department of Nephrology and Endocrinology II, Dongzhimen Hospital Affiliated to Beijing University of Chinese Medicine, Beijing, China, dzmhospital.com; ^2^ Department of Nephrology, Hangzhou Hospital of Traditional Chinese Medicine Affiliated to Zhejiang Chinese Medical University, Hangzhou, Zhejiang Province, China, hztcm.net; ^3^ Shandong University of Traditional Chinese Medicine, Jinan, Shandong Province, China, sdutcm.edu.cn; ^4^ Affiliated Hospital Of Shandong University Of Traditional Chinese Medicine, Jinan, Shandong Province, China, sdutcm.edu.cn

**Keywords:** diabetic nephropathy, ginseng, macrophage, pyroptosis, renal tubular epithelial cell, Rhizoma Coptidis

## Abstract

**Purpose:**

The objective of this study is to investigate the efficacy of the Shenlian (SL) decoction on regulating cellular pyroptosis and macrophage M1 polarization in the treatment of diabetic nephropathy (DN), and to elucidate its mechanism of action through the use of animal and cellular experiments.

**Methods:**

The potential targets for SL decoction were predicted using the TCMSP and Swiss Target databases. The differential genes for DN were obtained using the GeneCards database, and potential mechanisms for the treatment of DN with SL decoction were explored through enrichment analysis. The efficacy of SL decoction in the treatment of DN and its regulation of macrophage polarization and pyroptosis were evaluated in vivo using db/db mice. The RAW264.7 cell line and the TCMK‐1 cell line were cultured in vitro. The objective was to investigate the effect of SL decoction on the inhibition of M1 macrophage polarization and scorched death of renal tubular epithelial cells (TECs).

**Results:**

The results of the enrichment analysis indicated that the Toll‐like signaling pathway was a principal pathway in the treatment of DN with SL decoction. The results of animal and cellular experiments demonstrated that SL decoction has the potential to enhance renal function in patients with DN, while simultaneously reducing the concentration of serum inflammatory factors. Additionally, it was observed that SL decoction could inhibit the infiltration of macrophages and the polarization of macrophages into the M1 phenotype. Furthermore, the activation of the TLR4 signaling pathway and the pyroptosis of renal tubular cells were also inhibited by SL decoction.

**Conclusion:**

This study employed network pharmacology and in vivo and in vitro experiments to confirm that SL decoction can improve renal function in patients with DN. The results demonstrated that the mechanism of action of SL decoction is related to regulating the M1 polarization of macrophages in the DN kidney and inhibiting the pyroptosis of renal TECs.

## 1. Introduction

Diabetic nephropathy (DN) is one of the common complications of diabetes mellitus, which has a serious impact on the microvascular system and is the main cause of chronic kidney disease (CKD) worldwide. According to statistics, about 30%–40% of diabetic patients will develop DN, of which 5%–10% will eventually progress to end‐stage kidney disease (ESRD) [1]. Recent studies have shown that renal tubular epithelial cell (TEC) death and interstitial inflammation promote the development of DN, with pyroptosis being an important mechanism of TEC death. Pyroptosis is an inflammation‐induced cell death pathway that is characterized by cell swelling and blebbing, formation of pores within the cell membranes, activation of inflammatory vesicles, and release of proinflammatory cytokines [2]. Activation of NLRP3 inflammatory vesicles is an important pathway for inducing cellular pyroptosis, when activated, NLRP3 sensors recruit the articulatory molecule ASC, activate the protease caspase‐1, which trigger the Gasdermin D (GSDMD) to form membrane pores, and mediate the secretion of IL‐1β and IL‐18 leading to membrane leakage, then ultimately the flattening cell’s cytoplasm [3]. Inflammation is the main cause of induced pyroptosis, and TLR4 recognizes inflammatory factors, activates NF‐κB, and induces pyroptosis [4].

Macrophage plays a significant role in the immune response associated with DN. Macrophages within the kidney can be divided into two categories: resident macrophage and infiltrating macrophage. Resident macrophage can be activated rapidly during the initial stages of DN [5]. Concurrently, elevated glucose levels also stimulate the secretion of chemokines (CCL2 and CCL5) by TEC [6], which in turn induce the formation of infiltrating macrophage in the kidney by circulating monocytes. Macrophages can be classified according to their function into two main categories: pro‐inflammatory aka M1, and M2, characterized with its inhibitory inflammatory, pro‐fibrotic, and reparative effects [7]. It has been demonstrated that M1 macrophage are capable of activating NLRP3 inflammatory vesicles in TEC, which in turn results in TEC pyroptosis [8].

Shenlian (SL) decoction is an herbal formula composed of Rhizoma Coptidis and Ginseng. Prior research has demonstrated that SL decoction can regulate the relative abundance of intestinal flora and enhance serum LPS and IL‐1β levels in db/db mice, indicating that the SL decoction may mitigate inflammation in db/db mice by modulating intestinal flora [9]. The active ingredient of Rhizoma Coptidis, berberine, has been demonstrated to reduce NF‐κB activity [10] and inhibit the expression of NLRP3, caspase‐1, and GSDMD, thereby inhibiting cellular pyroptosis [11]. The active components of ginseng, ginsenosides Rg1 and Rg3, have been demonstrated to reduce the levels of PI3K, NF‐κB, and TNF‐α in DN rats [12–14]. Ginsenoside Rg5 has been demonstrated to inhibit cellular pyroptosis in diabetic kidney disease kidneys by reducing the expression of NLRP3, ASC, caspase‐1, and inflammatory factors (IL‐1β and IL‐18) [15]. The results of these studies collectively indicate that SL decoction may retard the progression of DN by inhibiting inflammatory processes, thereby slowing the progression of DN.

The present study is aimed to ascertain the efficacy of SL decoction in the management of DN. To this end, we conducted a series of animal and cellular experiments, which revealed that SL decoction exerts its renoprotective effects by inhibiting the TLR4 signaling pathway, regulating macrophage M1 polarization and inhibiting renal TEC pyroptosis.

## 2. Material and Methods

### 2.1. Network Pharmacology

We retrieved the active ingredients and chemical formulae of SL decoction through TCMSP (https://old.tcmsp-e.com/tcmsp.php) and Pubchem Server (https://pubchem.ncbi.nlm.nih.gov/). Pharmacokinetic filtration parameters were implemented with dual criteria: oral bioavailability (OB) ≥ 30% and drug‐likeness index (DL) ≥ 0.18. The target genes of the active ingredients were predicted using Swiss Target Prediction (http://www.swisstargetprediction.ch/) and the screening criterion was probability > 0. Disease‐associated targets for DN were retrieved from GeneCards (https://www.genecards.org/), employing “Diabetic nephropathy” as the query term, with subsequent filtration retaining entries demonstrating relevance scores >1. Therapeutic target exploration for SL decoction in DN management was conducted via the STRING platform (https://cn.string-db.org/). Intersection targets between SL decoction components and DN pathogenesis were analyzed under controlled parameters: organism restricted to *Homo sapiens* and interaction confidence cutoff ≥0.7 (except for text mining). Biological interpretation was achieved through Gene Ontology (GO) and Kyoto Encyclopedia of Genes and Genomes (KEGG) analyses using DAVID resources (https://david.ncifcrf.gov/). Analytical outcomes were graphically represented via the bioinformatics visualization portal (https://www.bioinformatics.com.cn/), delineating critical biological processes and signaling cascades.

### 2.2. Drugs

Rhizoma Coptidis and ginseng were procured from Beijing Kangmei Pharmaceutical Co., Ltd. The content of representative chemical components was determined by the Research and Experiment Center of Beijing University of Chinese Medicine, as previously documented in our extant literature [9]. The solution was filtered through double‐layered cotton gauze, and the filtrate was collected and combined to be decocted and concentrated until it contained the same concentration as the raw herb (4.3 g/kg). The solution was then stored at 4°C for future use.

The metformin hydrochloride tablets (0.5 g/tablet, Zhongmei Shanghai Schweppes Pharmaceutical Co., Ltd., National Drug Code H20023370) were weighed at 1.5 g and subsequently crushed. The metformin hydrochloride suspension was prepared by dissolving the drug in distilled water to a concentration of 19.5 mg/mL. The suspension was stored at 4°C and used within 60 min at room temperature, with agitation to ensure uniformity. The suspension was prepared once a week.

### 2.3. Animal Experiments

A total of 18 8‐week‐old male db/db (C57BLKS) mice, a spontaneous type 2 diabetic strain, were purchased from Changzhou Cavins Laboratory Animal Co. These mice were of the same litter and had a body mass of 35–40 g. Additionally, 20–25 g wild‐type db/m mice from the same litter were also purchased. The mice were housed in the SPF‐class environmental barrier animal laboratory of Dongzhimen Hospital, Beijing University of Traditional Chinese Medicine (SYXK(Beijing)2020‐0013), with a 12‐h light/dark cycle. The animals were provided with a regular diet and drinking water. The basal diet was formulated as 65% sugar and water compounds, 24.2% protein and 10.3% fat, which was supplied by Beijing Huafukang Bio‐technology Co., Ltd. (No. 1022). The animal experiment protocol was approved by the Animal Welfare Committee of Beijing University of Traditional Chinese Medicine (No. 21–19). At 10 weeks of age, the mice were subjected to a blood collection procedure via the tail vein, and their fasting blood glucose levels were quantified in order to confirm the presence of spontaneous hyperglycemia. A blood glucose level exceeding 16.7 mM is indicative of diabetes. Starting at week 10, db/db mice were randomly assigned to one of three groups: a distilled water model group (10 mL/kg), a metformin‐treated group (0.195 g/kg), and an SL decoction‐treated group (4.3 g/kg SL decoction). The normal group consisted of six db/m mice. All treatments were administered by gavage for 9 weeks. Mouse dose (g/kg) = 9.1 × human clinical dose (g/kg). Tail vein blood glucose levels were measured every 2 weeks. In addition, urine samples were collected from the mice every 3 weeks using metabolic cages to test urinary creatinine and urinary albumin levels. When the drug was administered by gavage for 9 weeks, after drug intervention, mice were fasted for 12 h, then anesthetized with 3% (1.6 mL/kg) pentobarbital sodium solution, and finally samples were collected.

SD rats were purchased from Cavins Laboratory Animal Co.A total of 20 SD rats were randomly assigned to one of two treatment groups: 10 received SD tonics by gavage and 10 received distilled water by gavage. All treatments were administered twice daily for 7 days. Following a 12‐h fast prior to the final administration, blood was collected via aseptic technique from the abdominal aorta, and subsequently centrifuged at 3000 rpm for 15 min 2 h post‐administration. Following a period of rest, the serum was separated and subsequently filtered and decontaminated through a 0.22 μm microporous filtration membrane. This process was employed to obtain the blank serum and the drug‐containing serum, which were then frozen at −20°C for future use.

### 2.4. Cell Culture

The cell line was obtained from the Cell Resource Center, Peking Union Medical College (which is part of the National Science and Technology Infrastructure, the National Biomedical Cell‐Line Resource, NSTI‐BMCR. http://cellresource.cn) on May 15, 2023. Mouse renal TEC line TCMK‐1 was purchased from Wuhan Xavier (STCC20015P‐1). Cell culture was performed in DMEM medium (DMEM; Pricella, China) containing 10% endotoxin‐free fetal bovine serum (FBS) that had undergone complement removal. All cells were cultured at 37°C and 5% CO_2_ for subsequent experiments.

### 2.5. Indicators of Biochemical and Oxidative Stress

Following anesthesia, the eyeballs were removed from the mice and the blood was collected. The blood was left at room temperature for 1 h and then centrifuged at 3000 rpm for 15 min. The supernatant was extracted and analyzed with reference to creatinine assay kit (C011‐1, Nanjing JianCheng, Nanjing, China), BUN assay kit (C013‐2, Nanjing Jianjian, Nanjing, China), malondialdehyde (MDA) colorimetric assay kit (E‐BC‐K025‐M, Elabscience, Wuhan, China), total superoxide dismutase (T‐SOD), Catalase (CAT) colourimetric test kit (No. E‐BC‐K031‐M, Elabscience, Wuhan, China), Interleukin 1β (IL‐1β) ELISA kit (E‐MSEL‐M0003, Elabscience, Wuhan, China), Interleukin 6(IL‐6) ELISA Kit (E‐MSEL‐M0001, Elabscience, Wuhan, China), Tumor Necrosis Factor Alpha (tumornecrosisfactorα, TNF‐α) ELISA Kit (E‐MSEL‐M0002, Elabscience.Wuhan, China) instruction steps for the assay.

### 2.6. Determination of Urinary Protein Index

The 6‐h urine samples from each group collected in the ninth week of drug intervention were subjected to centrifugation at 3000 rpm (centrifugal radius 8 cm) at 4°C for 15 min. The volume of urine was calculated, and the supernatant was transferred for the detection of urinary creatinine and microalbumin. The urinary microalbumin kit (Item No. ML063626‐2, Shanghai Enzyme‐Link Bio‐Technology Co., Ltd.) was used to determine the albumin/urine creatinine ratio (ACR) and 6 h urine total protein quantity (6hUTP).

### 2.7. Renal Pathology

The mouse kidney tissues were fixed in 4% paraformaldehyde. Following routine dehydration and embedding, 4‐μm sections were obtained and subjected to a series of staining techniques, including hematoxylin–eosin (H–E), Masson trichrome, and periodate‐Schiff staining (PAS). The H–E staining solution (D006), Masson’s staining solution (D026‐1‐2), and Aisin blue‐glycogen staining solution (D033‐1‐1) were procured from the Nanjing Jiancheng Bioengineering Institute. The staining was carried out in the Key Discipline Laboratory of Beijing University of Chinese Medicine according to the manufacturer’s instructions. The images for subsequent analysis were obtained using a bx51 optical microscope (OLYMPUS, Japan).

### 2.8. Immunohistochemistry (IHC) and Immunofluorescence (IF)

For IHC, tissue sections were subjected to routine antigen repair, incubated with 3% hydrogen peroxide to block endogenous peroxidase activity, sealed with BSA, incubated overnight with TLR4 antibody (1:2000; Santa, ab92726), p‐NF‐κB p65 (1:2000; sc‐136548, Santa), NF‐κB p65 antibody (1:2000; A2547, ABclonal), GSDMD‐N antibody (1:1000; CQA6563, cohesion), caspase‐1 antibody (1:2000; CPA5426, cohesion), NLRP3 antibody (1:2000; bs‐6655, Bioss), IL‐18 antibody (1:2000; YN1926, immunoway), IL‐1β (1:2000; sc‐52012, Santa), F4/80 (1:1000; SC‐377009, Santa) incubation overnight, rinsed three times with PBS, incubated with the corresponding secondary antibodies, and treated with DAB for color development. Following re‐staining of the nuclei, the sections were sealed with neutral balsam and images were obtained using a Leica microscope.

For IF, tissue sections were antigenically repaired, closed with 3% hydrogen peroxide at room temperature, closed with BSA, and incubated overnight at 4°C with primary antibodies CD68 (1:200; 28058‐1‐AP, ProteinTech) and NOS2 (1:500; SC‐7271, Santa). Following three washes with PBS, the sections were incubated with the corresponding secondary antibodies, washed three times with PBS, and treated with CY3‐Tyramine Signal Amplification (TSA) and/or FITC‐TSA. Cell nuclei were restained with DAPI, the sections were sealed using an antifluorescence quencher, and images were taken under a Leica fluorescence microscope.

### 2.9. Western Blots

Cells were lysed on ice for 30 min using RIPA lysis buffer (C1053, Applygen) containing protease inhibitor (GRF101, Yase), phosphatase inhibitor (GRF102, Yase). The lysate was then transferred to a 1.5 mL centrifuge tube and centrifuged at 12,000 rpm for 10 min at 4°C and the supernatant was collected. After protein concentration was determined using the BCA Protein Concentration Assay Kit (B5000, LabLead), 30 μg of tissue lysate was mixed with 5x SDS‐PAGE Denaturing Protein Supersample Buffer (B1012, Applygen).Samples were loaded on a 10% polyacrylamide gel, separated by SDS‐PAGE and transferred to a NC membrane (Millipore No. HATF00010). After closure with 5.0% milk containing 1% tween, primary anti‐TLR4 antibody (1:2000; sc‐293072, Santa), p‐NF‐κB p65 (1:2000; sc‐136548, Santa), NF‐κB p65 antibody (1:2000; A2547, ABclonal), GSDMD‐N antibody (1:1000; CQA6563, cohesion), caspase‐1 antibody (1:2000; CPA5426, cohesion), NLRP3 (1:2000;bs‐6655R, Bioss), β‐actin antibody (1:2500; ab9485, Abcam), Histone H3 antibody (A2348,. ABclonal) were added and incubated at 4°C overnight. After adding HRP Goat Anti‐Rabbit IgG(H+L) (AS014, ABclonal) or HRP Goat Anti‐Mouse IgG(H+L) (AS003, ABclonal) and incubating for 2 h at room temperature, ECL chemiluminescent substrate (extra‐ultrasensitive) (biosharp BL520A) was added dropwise to the membrane and placed into a Gel Doc imaging system (Bio‐Rad) for photographing. Semi‐quantitative analyses were performed using ImageJ software.

### 2.10. RNA Extraction and Real‐Time Quantitative Polymerase Chain Reaction (qRT‐PCR)

Total RNA was extracted from animal tissues and cells using the AFTSpin tissue/cell fast RNA extraction Kit for animal (RK30120, ABclonal) according to the manufacturer’s instructions. Reverse transcription reactions were performed using the ABScript III RT Master Mix for qPCR with gDNA Remover kit (RK20429; ABclonal). We constructed specific primers (Table 1, Primer sequence) and performed real‐time fluorescence quantitative PCR using 2x Universal SYBR Green Fast qPCR Mix (RK21203, ABclonal). Relative expression levels were calculated using the 2‐delta delta CT method, and each set of experiments was repeated more than three times.

**Table 1:** Primer sequence of qRT‐PCR.

Primers	Forward primer	Reverse primer
NLRP3	ATCAACAGGCGAGACCTCTG	GTCCTCCTGGCATACCATAGA
GSDMD	TTCCAGTGCCTCCATGAATGT	GCTGTGGACCTCAGTGATCT
Caspase‐1	CTTGGAGACATCCTGTCAGGG	AGTCACAAGACCAGGCATATTCT
TLR4	GCCTTTCAGGGAATTAAGCTCC	GATCAACCGATGGACGTGTAAA
NF‐KB P65	ACTGCCGGGATGGCTACTAT	TCTGGATTCGCTGGCTAATGG
Arg‐1	TGTCCCTAATGACAGCTCCTT	GCATCCACCCAAATGACACAT
NOS2	GGAGTGACGGCAAACATGACT	TCGATGCACAACTGGGTGAAC
β‐actin	GTGACGTTGACATCCGTAAAGA	GCCGGACTCATCGTACTCC

### 2.11. Statistical Analysis

Data with a normal distribution are expressed as mean ± standard deviation. Data from multiple groups were compared using one‐way ANOVA, and Fisher’s least significant difference test was performed for multiple comparisons of differences between groups. Differences were considered significant at *p*  < 0.05. SPSS 23.0 statistical software was used for analysis.

## 3. Results

### 3.1. A Network Pharmacological Analysis Has Revealed that SL Decoction Regulated Targets Are Enriched in the TLR4 Pathway

Through the analysis of TCMSP database and related literature, 36 kinds of main active components were determined from the SL decoction. After removing duplicates, a total of 700 potential targets for the active ingredients were found, and they were subjected to screening (Supporting Information 1: Table S1). A search of the GeneCards platform for genes related to DN returned with 4050 results (Supporting Information 2: Table S2). By mapping the cross‐targets of the SL decoction active ingredients to the disease targets of NA, a total of 343 cross‐targets were identified (Figure 1A). Subsequently, the PPI network was constructed for the purpose of identifying key genes (Figure 1B). A GO enrichment analysis conducted via the DAVID database (Figure 1C, Supporting Information 3: Table S3) revealed that BPs are predominantly involved in protein phosphorylation, protein autophosphorylation, and response to xenobiotic stimulus. CCs are primarily concentrated in the integral component of the plasma membrane, the plasma membrane itself, and the cytosol. Microtubule‐associated proteins (MAPs) are primarily associated with the activities of serine/threonine/tyrosine kinases, protein kinases, and ATP binding. Furthermore, a KEGG enrichment analysis was conducted (Figure 1D, Supporting Information 4: Table S4). Following the removal of pathways that are unrelated to DN, the most significant enriched pathways were identified as the PI3K‐Akt signaling pathway, the chemokine signaling pathway, and the Toll‐like receptor signaling pathway. The Toll‐like receptor signaling pathway was subjected to analysis, which revealed that the target genes of SL decoction predominantly encompass TLR4, NF‐κB and additional genes (Figure 1E).

Figure 1Venn diagrams of target genes of SL decoction with DN (A); PPI networks based on SL decoction potential targets (B); GO enrichment analysis of candidate targets (C); KEGG enrichment analysis of candidate targets (D); Relative location of potential SL decoction targets in Toll‐like signaling pathways (E).

**Figure:**
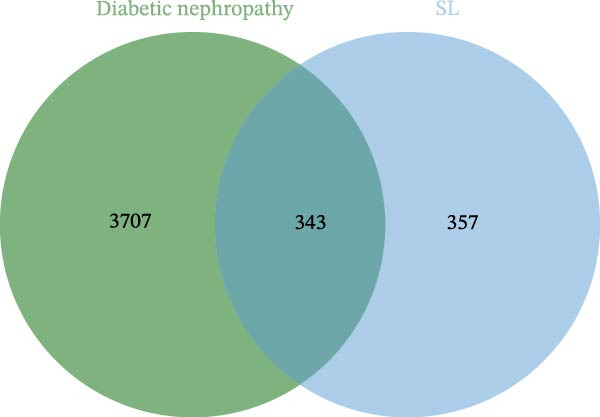
(A)

**Figure:**
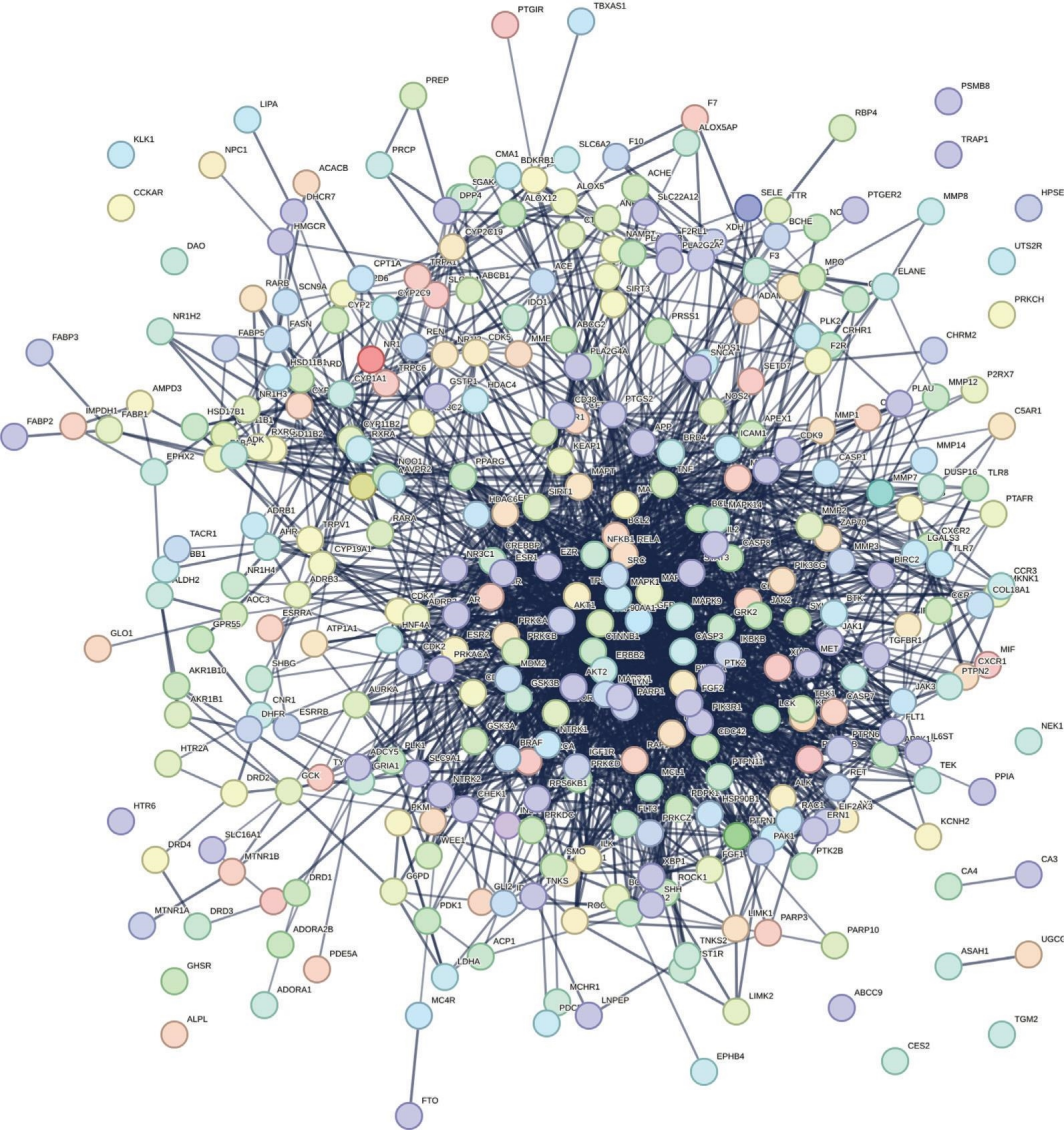
(B)

**Figure:**
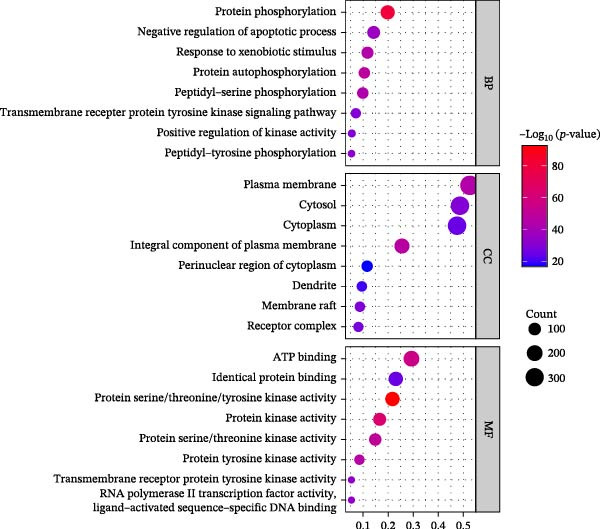
(C)

**Figure:**
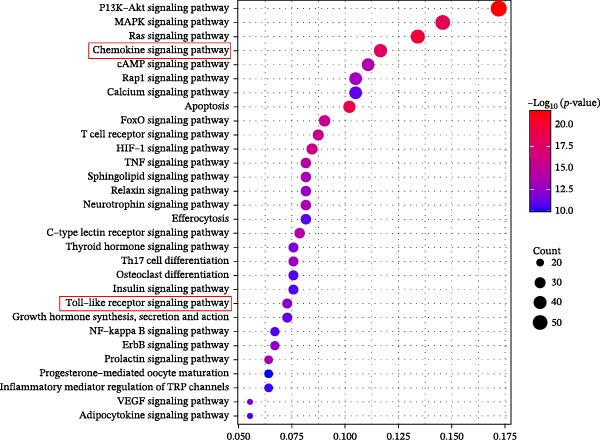
(D)

**Figure:**
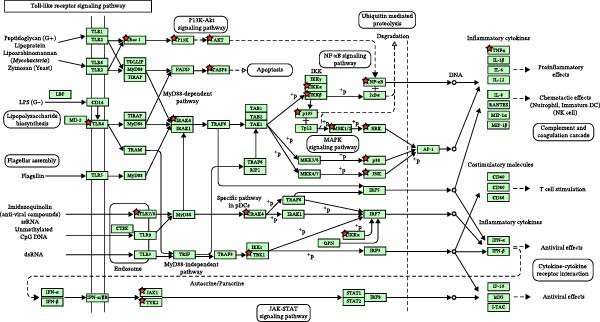
(E)

### 3.2. SL Decoction Improved Renal Function and Renal Pathology in db/db Mice

We used db/db mice, a model of spontaneous type 2 diabetes, for our study. At 8 weeks of age, db/db mice exhibited significantly elevated blood glucose levels compared to their normal control counterparts, accompanied by a notable surge in UACR (Figure 2A,B). The administration of metformin and SL decoction on a weekly basis via gavage was conducted for a period of 9 weeks. Following the administration of metformin and SL decoction, mice exhibited reduced blood glucose and ACR levels in comparison to the DN group. However, no statistically significant differences were observed (Figure 2A,B). Following a 9‐week administration period, serum samples were collected from the mice for testing. The results demonstrated a significant reduction in Scr and BUN levels in the administered group compared to the DN group (Figure 2C,D). Additionally, the efficacy of the SL decoction group was comparable to that of the metformin group.

Figure 2Fasting blood glucose (*n* = 6) (A); ACR (*n* = 6) (B); Serum creatinine (*n* = 6) (C); urea nitrogen (*n* = 6) (D); H–E dyeing (×200), PAS dyeing (×400), Masson dyeing (×200) (E).

**Figure:**
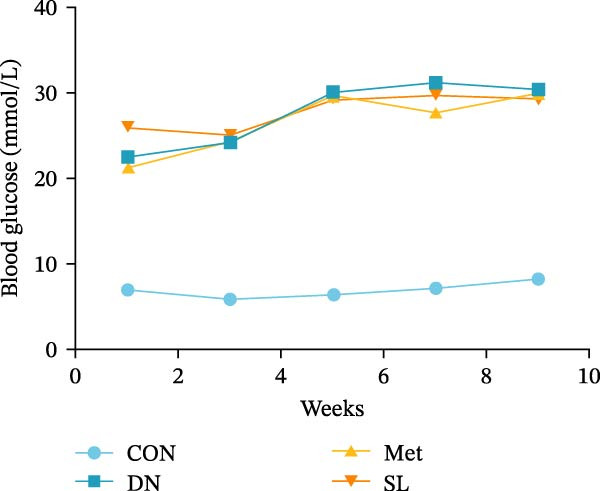
(A)

**Figure:**
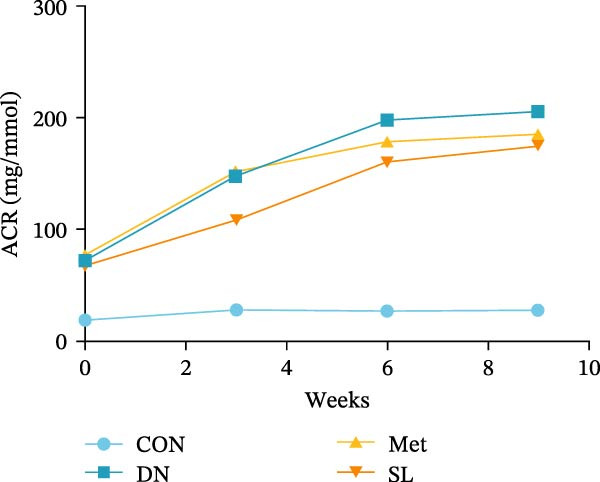
(B)

**Figure:**
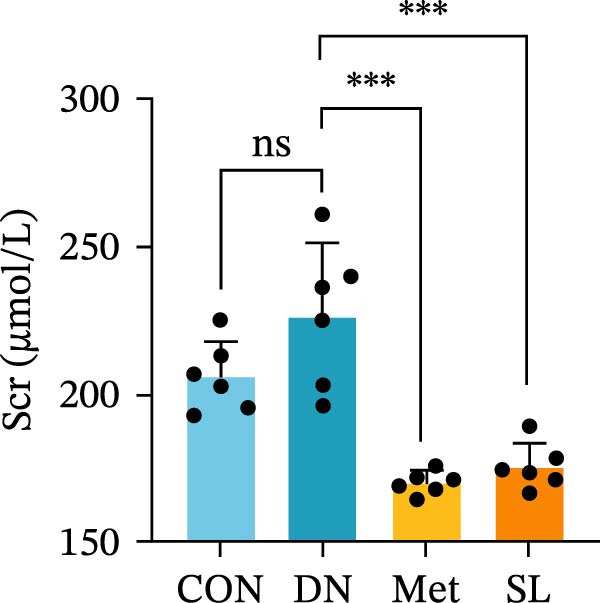
(C)

**Figure:**
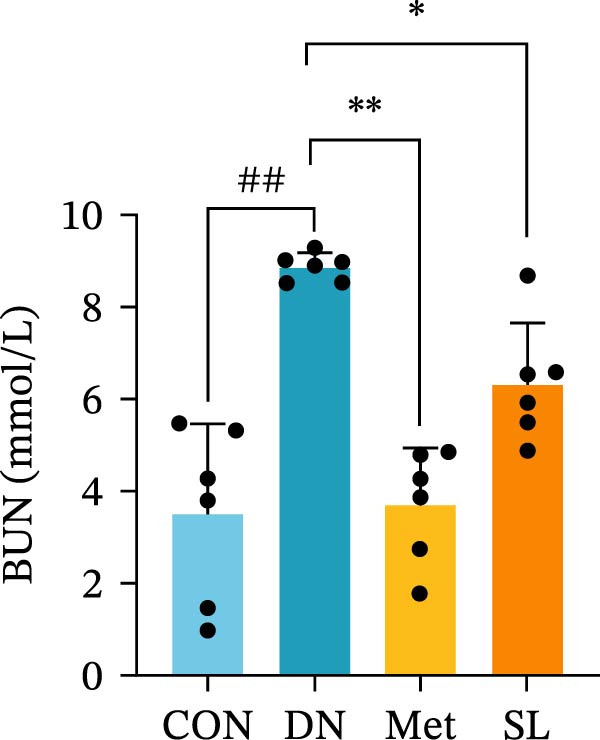
(D)

**Figure:**
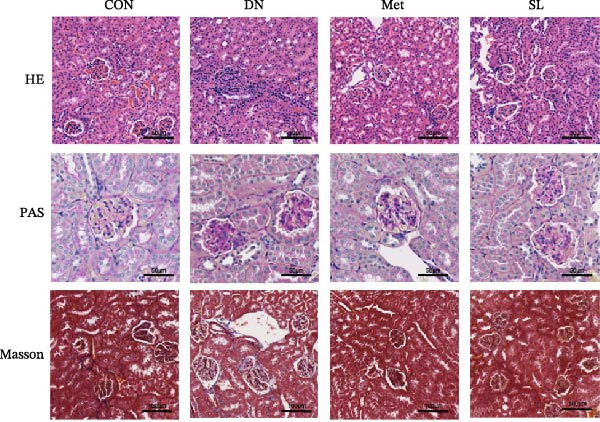
(E)

Concurrently, SL decoction demonstrated a marked capacity to mitigate the pathological deterioration of renal tissue in mice afflicted with DN. The objective of this study was to investigate the effect of SL decoction on histopathological damage in diabetic kidneys. Kidney samples were collected from each group of mice and stained with H–E, PAS, and Masson for pathological analysis. The results demonstrated that the kidneys of mice in the model group exhibited glomerular hypertrophy, dilated and congested capillary collaterals, proliferation of mesangial cells, dilated mesangial stroma, narrowed renal capsule, blurred luminal rim of renal tubules, congested and edematous renal interstitium, and a notable increase in the infiltration of inflammatory cells, in comparison to those of the blank control group (Figure 2E; H–E staining). PAS staining revealed that the kidneys of mice in the model group exhibited dilated capillary collaterals, proliferated mesangial cells, and markedly dilated thylakoid stroma in comparison to the blank control group (Figure 2E; PAS staining). The results of the Masson staining also demonstrated that the kidneys of the mice in the model group exhibited hyperplasia of the mesangial stroma, thickening of the glomerular basement membrane, and increased fibrosis in comparison to the blank control group (Figure 2E; Masson staining). The aforementioned pathological alterations exhibited a notable improvement in the metformin and SL decoction groups.

### 3.3. SL Decoction Improved Macrophage Infiltration and M1 Polarization in db/db Mice

To further investigate the M1 polarization of macrophages in mouse kidneys, we performed IF co‐staining of paraffin sections of mouse kidneys with F4/80 for IHC and CD68/iNOS. This was done in order to detect infiltrating macrophages in mouse kidneys and to determine their polarization status. The DN group exhibited a notable elevation in F4/80+ macrophages infiltrating the glomerular and tubular regions (Figure 3A,B), accompanied by a substantial increase in the number of positive cells co‐localized with CD68/iNOS (Figure 3C,D). The intervention in the SL decoction and metformin groups resulted in a notable reduction in F4/80 and CD68/iNOS expression. These results indicate that SL decoction may play a role in reducing the infiltration of macrophages into the DN area and the polarization of macrophages to the M1 phenotype.

Figure 3IHC image of F4/80 (*n* = 6) (A,B); IF co‐localization image of iNOS/CD68 (*n* = 6) (C,D); Serum IL‐6, IL‐1β, TNF‐α levels (*n* = 6) (E); Serum MDA level (*n* = 6) (F); Serum SOD level (*n* = 6) (G); Serum CAT level (*n* = 6) (H).

**Figure:**
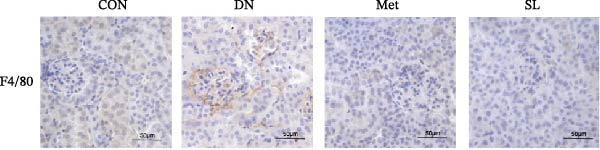
(A)

**Figure:**
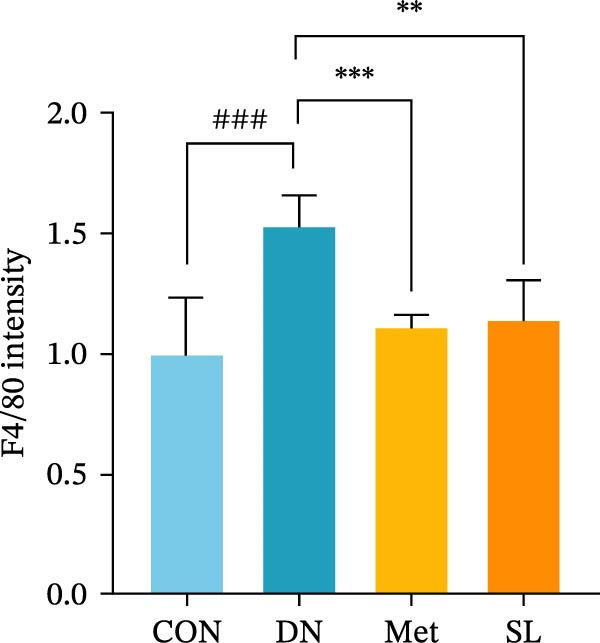
(B)

**Figure:**
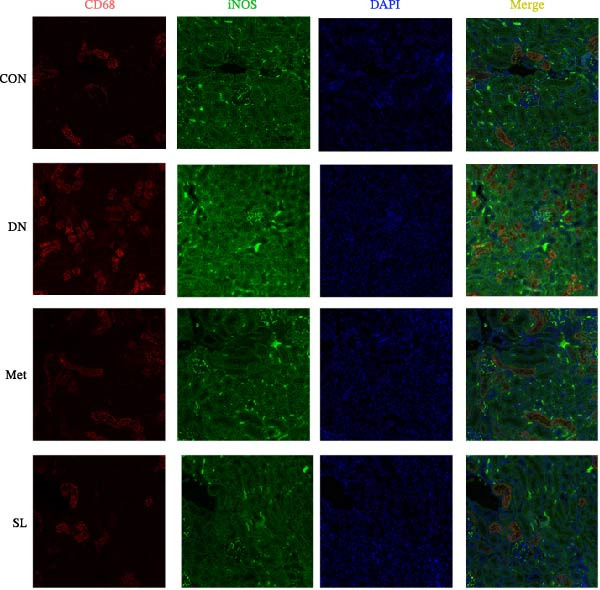
(C)

**Figure:**
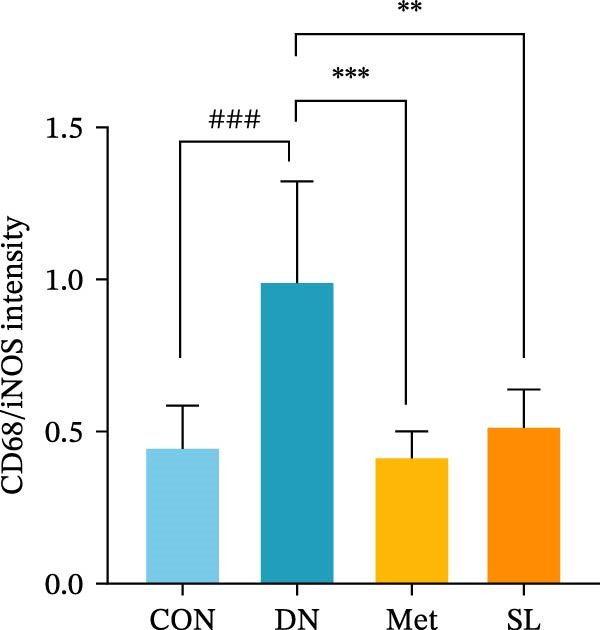
(D)

**Figure:**
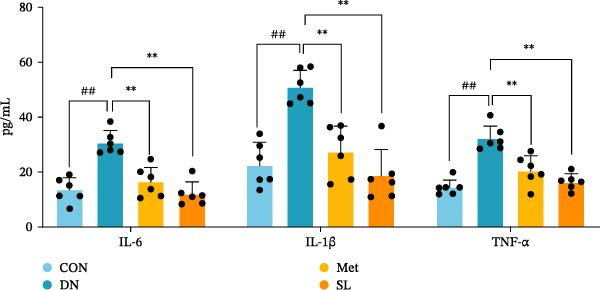
(E)

**Figure:**
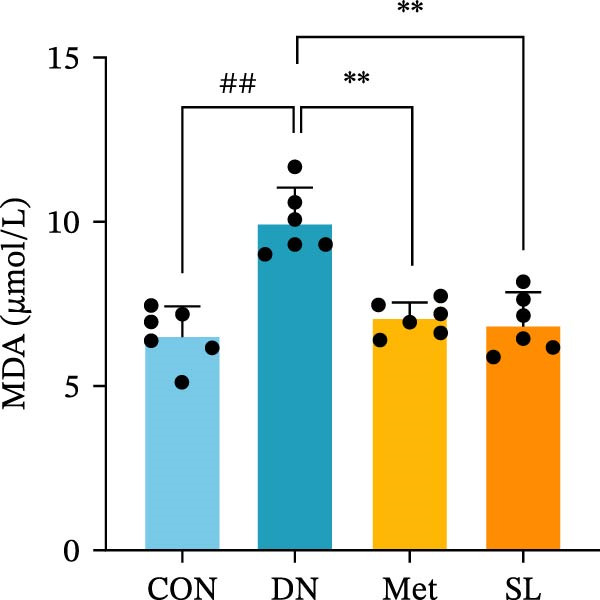
(F)

**Figure:**
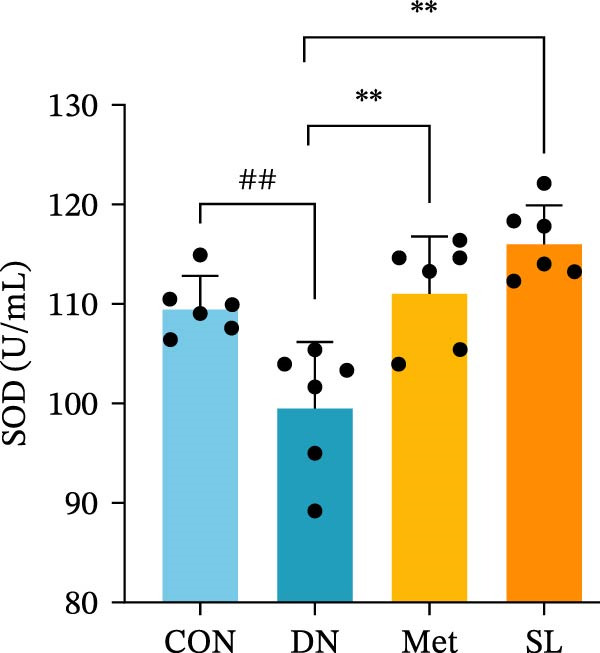
(G)

**Figure:**
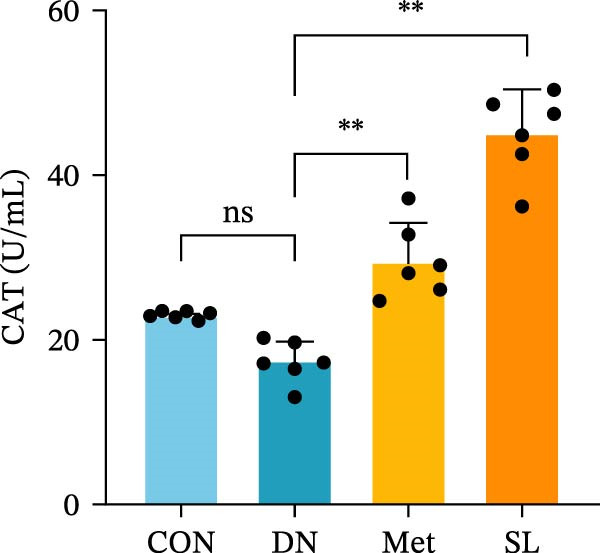
(H)

To further investigate the impact of macrophage infiltration in renal tissues on the inflammatory response, we conducted an enzyme‐linked immunosorbent assay (ELISA) for the inflammatory factors IL‐6, IL‐1β, and TNF‐α in mouse serum. The levels of inflammatory factors were markedly elevated in the DN cohort relative to the control group. Following SL decoction treatment, a notable reduction in serum inflammatory factor levels was observed (Figure 3E), with an efficacy comparable to that of metformin. Concurrently, we investigated the oxidative stress indicators SOD, CAT, and MDA in the serum of mice. In comparison to the control group, the DN group exhibited elevated levels of SOD and MDA, while CAT levels were diminished. Following SL decoction treatment, there was a notable decline in serum SOD and MDA levels, accompanied by a considerable increase in CAT (Figure 3F,G). These findings indicate that macrophage infiltration and M1 polarization may contribute to renal inflammatory infiltration in DN, and that SL decoction treatment may improve renal inflammatory state and oxidative stress.

### 3.4. SL Decoction Has Been Demonstrated to Inhibit the Activation of the TLR4 Signaling Pathway and Cellular Pyroptosis in db/db Mice

IHC staining of paraffin‐embedded mouse kidney sections revealed a significant elevation in the levels of TLR4, NF‐κB p65, and p‐NF‐κB p65 in the DN group. Conversely, SL decoction treatment resulted in a notable reduction in the levels of TLR4, NF‐κB p65, and p‐NF‐κB p65 in renal tissues (Figure 4A–D). It was proposed that SL decoction may inhibit the activation of the renal TLR4 signaling pathway in db/db mice. IHC of paraffin‐embedded sections of mouse kidneys revealed a significant elevation in caspase‐1, IL‐18, and IL‐1β levels in renal tubular cells within the DN group (Figure 4E–G). Furthermore, SL decoction treatment was observed to significantly reduce renal tissue caspase‐1, IL‐18, and IL‐1β levels. The results of the WB analysis demonstrated that the pyroptosis‐related proteins GSDMD, GSDMD‐NT, Caspase‐1, and NLRP3 were expressed and exhibited a significant increase in the diabetic kidneys. The levels of pyroptosis‐associated proteins in renal tissues were significantly diminished following SL decoction treatment (Figure 4H–L).

Figure 4IHC of TLR4, NF‐κB p65, p‐NF‐κB p65, caspase‐1, IL‐1β, and IL‐18 (A); Relative intensity of TLR4, NF‐κB p65, p‐NF‐κB p65, caspase‐1, IL‐1β, and IL‐18 in the kidney tissues (*n* = 6) (B–G); Western blots of NLRP3, Caspase‐1, and GSDMD (*n* = 3) (H–L).

**Figure:**
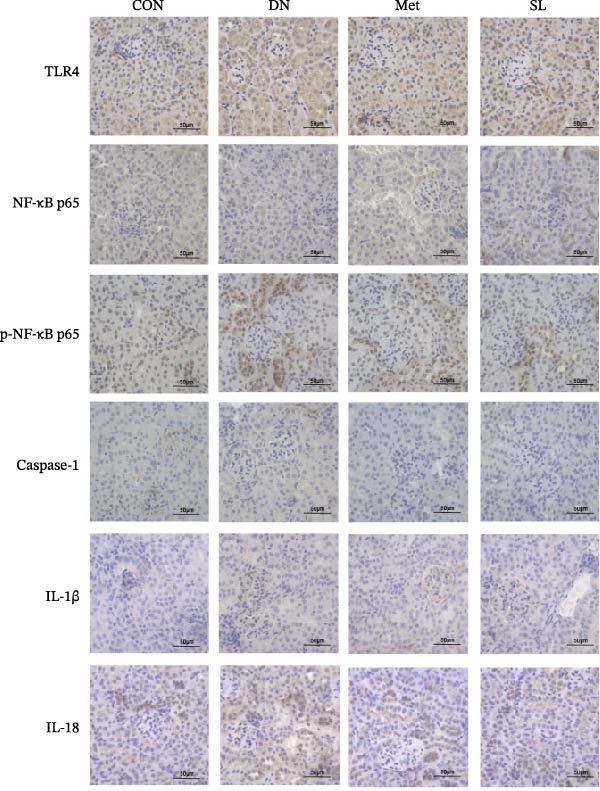
(A)

**Figure:**
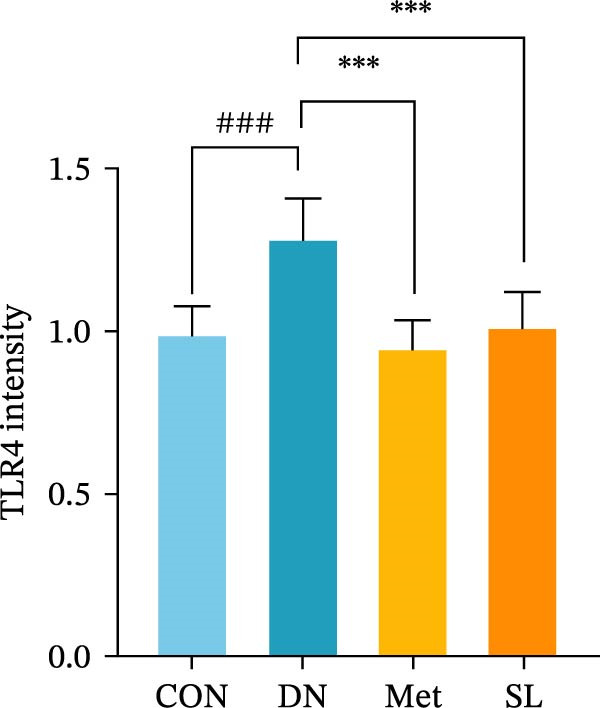
(B)

**Figure:**
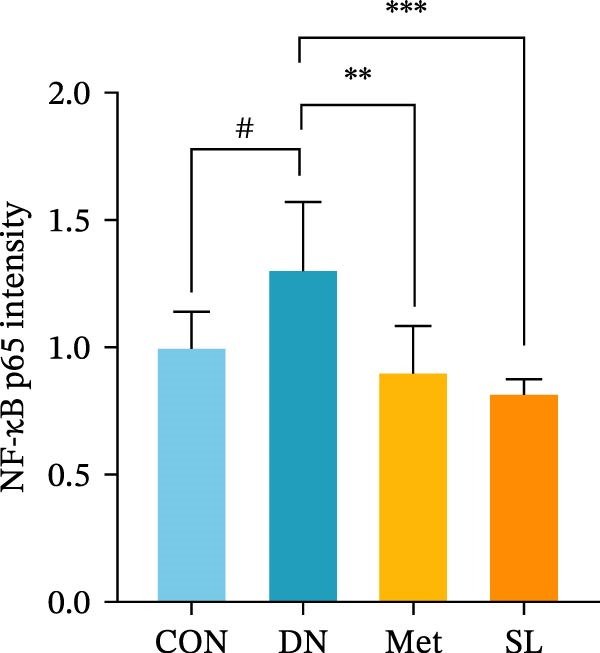
(C)

**Figure:**
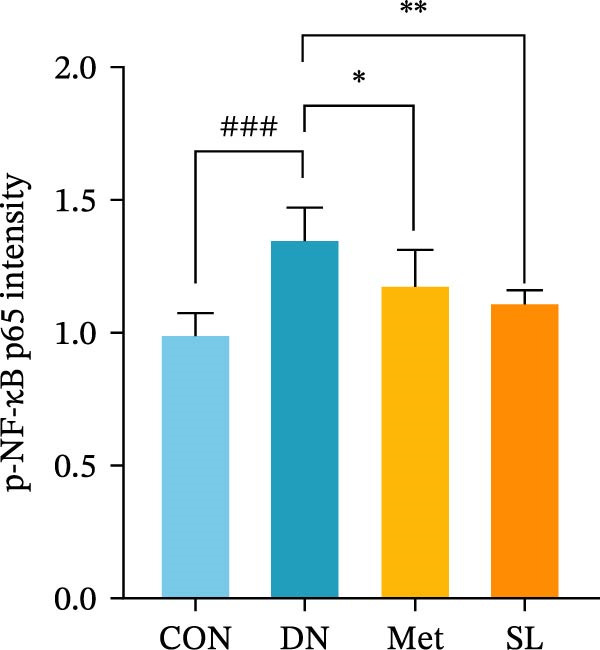
(D)

**Figure:**
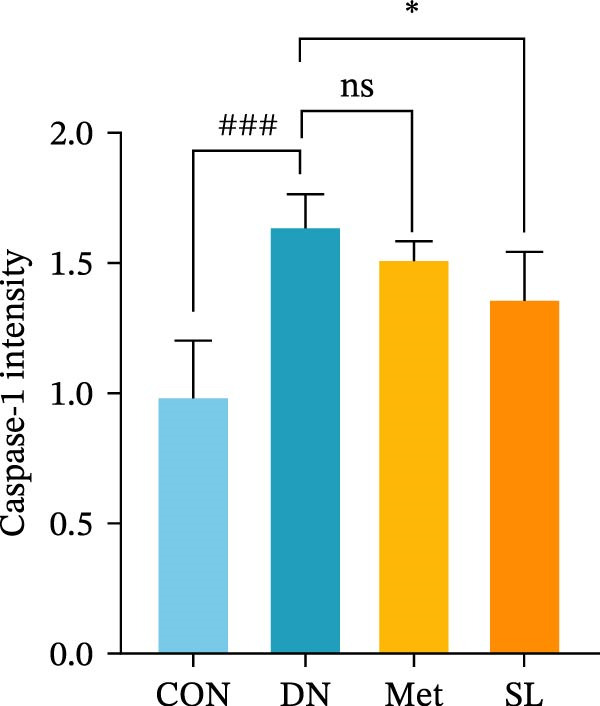
(E)

**Figure:**
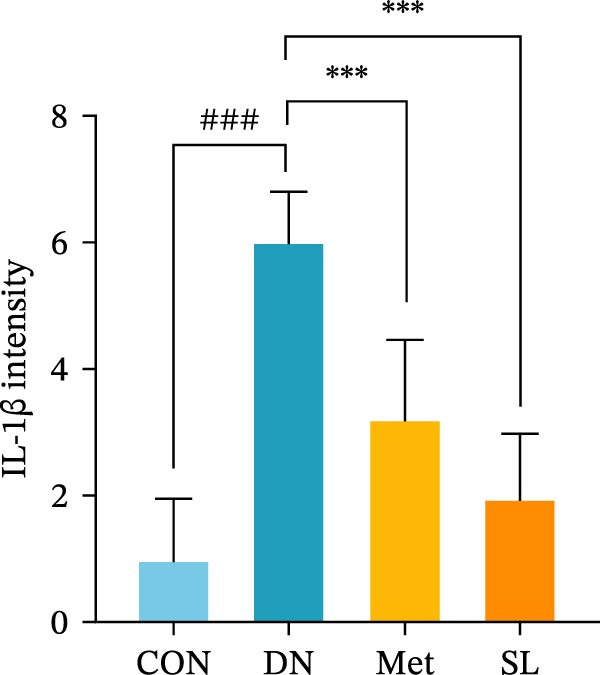
(F)

**Figure:**
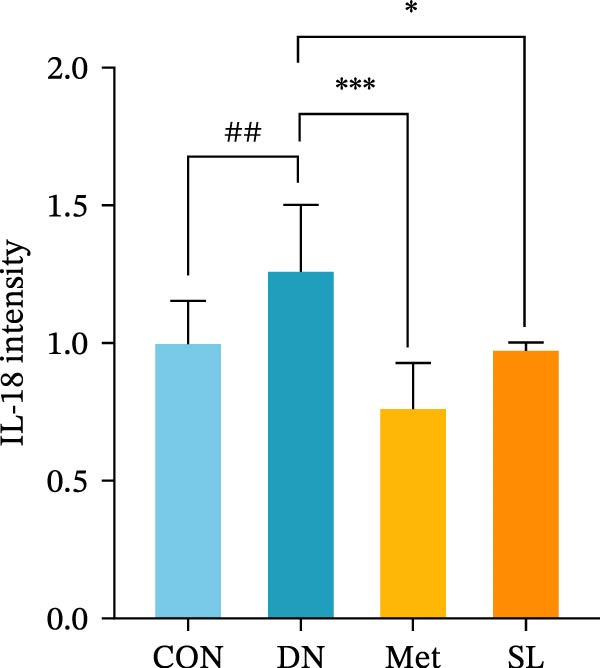
(G)

**Figure:**
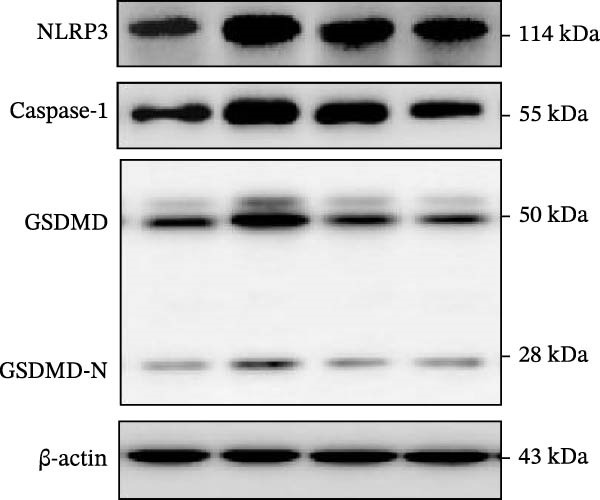
(H)

**Figure:**
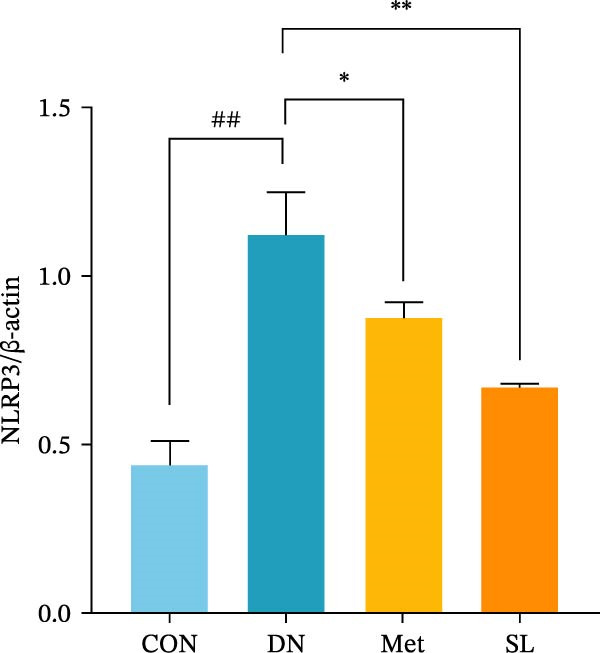
(I)

**Figure:**
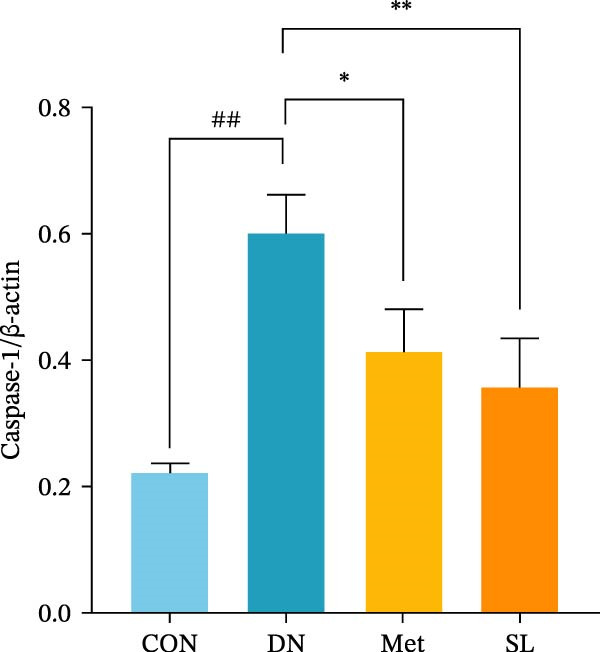
(J)

**Figure:**
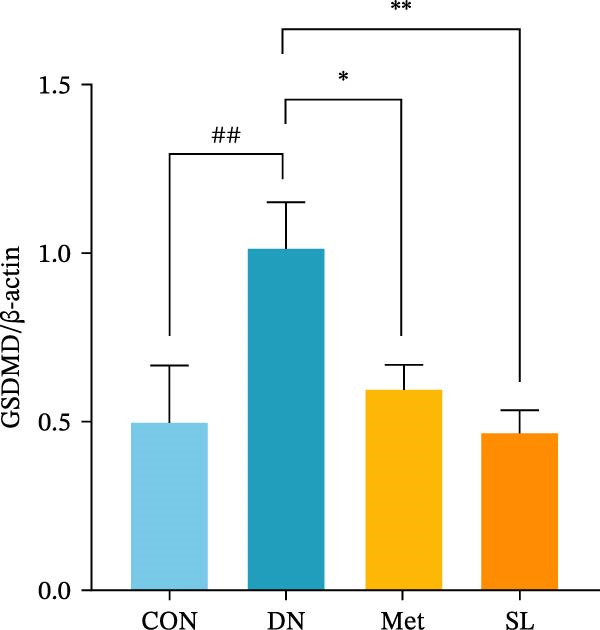
(K)

**Figure:**
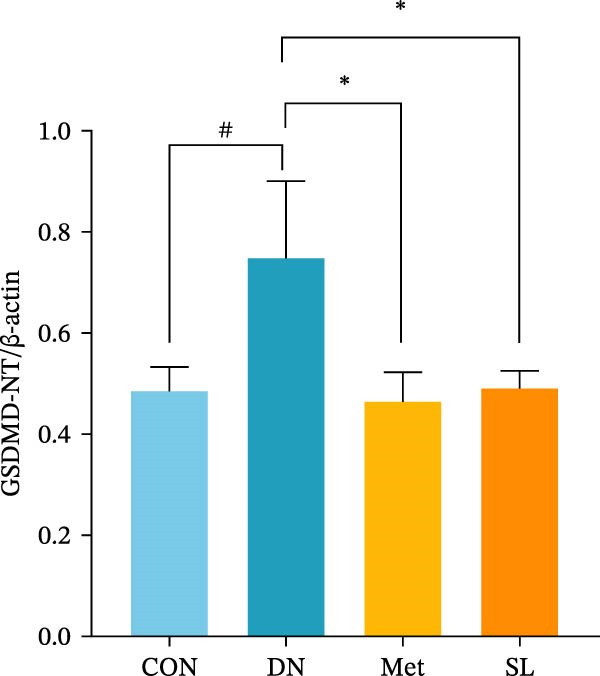
(L)

### 3.5. SL Decoction Has Been Demonstrated to Inhibit Macrophage M1 Polarization by Modulating the TLR4 Pathway

The TLR4 signaling pathway plays a significant role in the polarization and maintenance of macrophage phenotypes. To further investigate whether SL decoction regulation of M1 polarization in DN kidney macrophage is associated with the TLR4 pathway, we conducted in vitro experiments using the mouse macrophage cell line RAW264.7. Following stimulation of the TLR4 pathway with LPS, there was an increase in TLR4 expression and NF‐κB p65 nuclear translocation, which was inhibited by the intervention of SL decoction (Figure 5A–E). The intervention of SL decoction resulted in a notable shift from M1 to M2 polarization in macrophages, as evidenced by a significant decrease in iNOS expression, an increase in Arg‐1 expression, a notable decline in the pro‐inflammatory factor IL‐6 in the cellular supernatant, and a substantial increase in the anti‐inflammatory factor IL‐10 (Figure 5F–J).

Figure 5Western blot images of NF‐κB p65 in nucleus and cytoplasm (*n* = 3) (A–C). The mRNA level of TLR4, NF‐κB p65, iNOS, Arg‐1 (*n* = 3) (D–G). IL‐6 and IL‐10 levels in cell supernatants (*n* = 3) (H, I).

**Figure:**
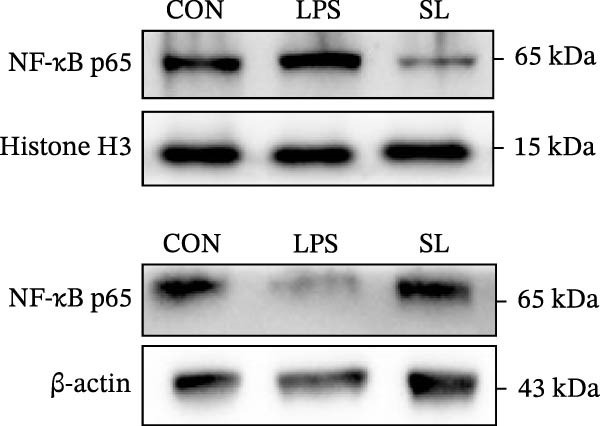
(A)

**Figure:**
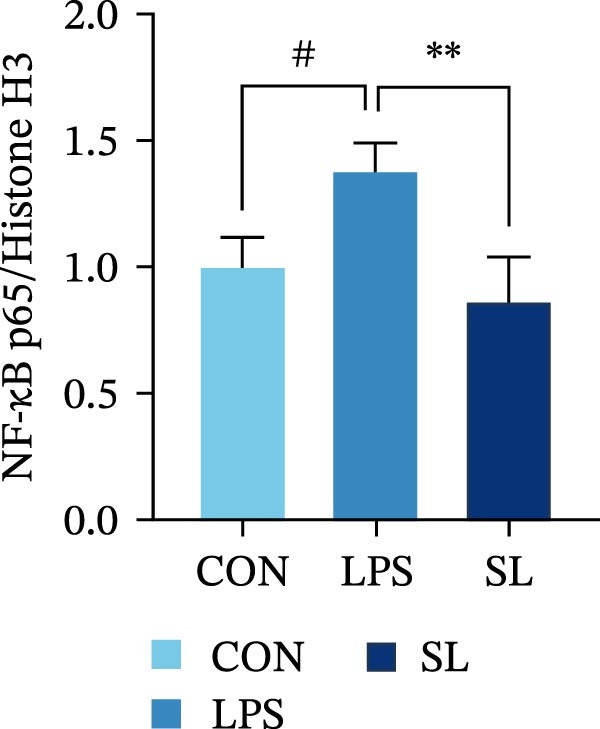
(B)

**Figure:**
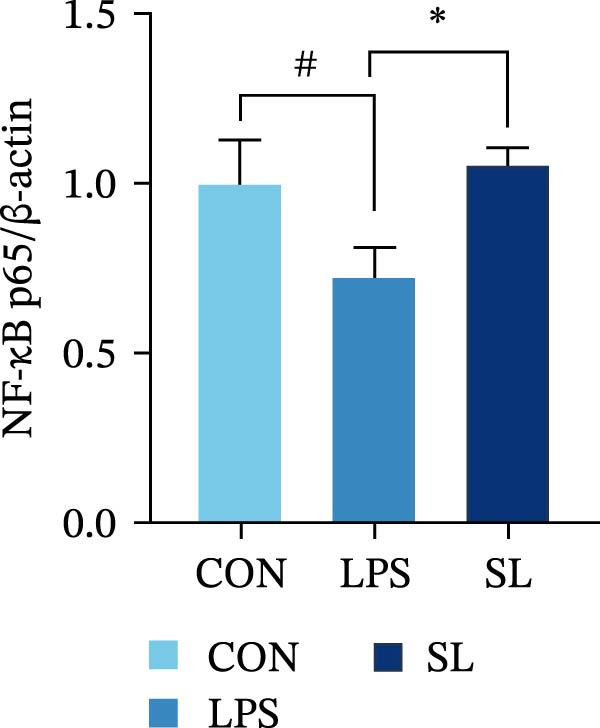
(C)

**Figure:**
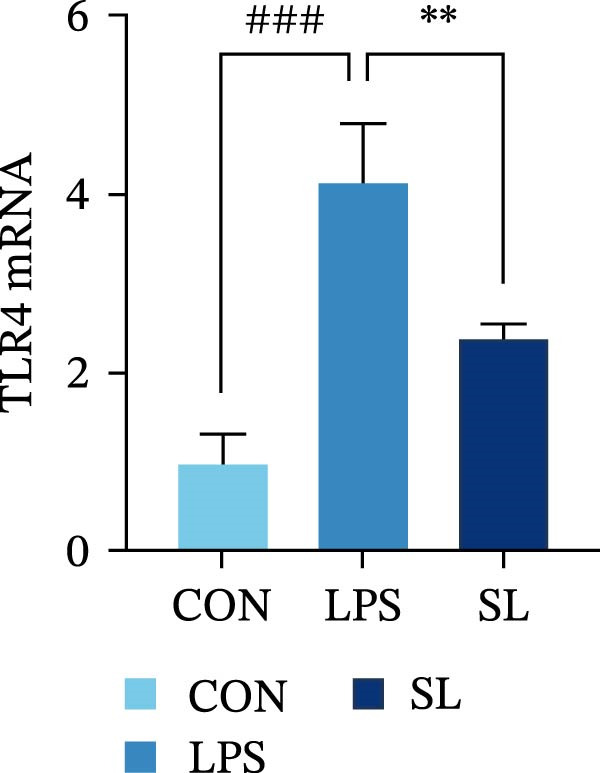
(D)

**Figure:**
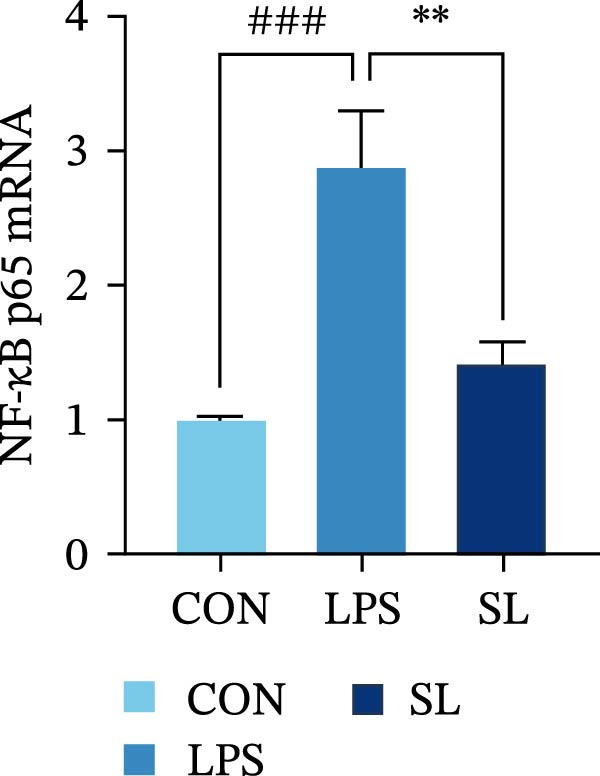
(E)

**Figure:**
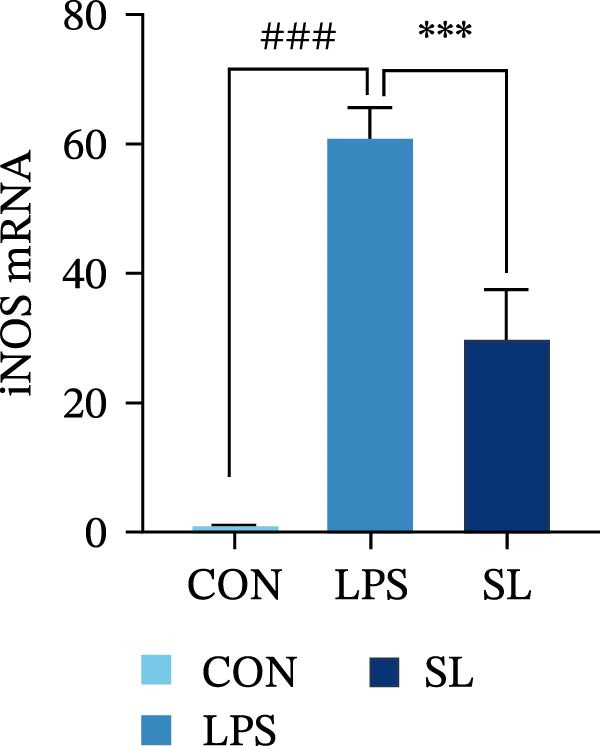
(F)

**Figure:**
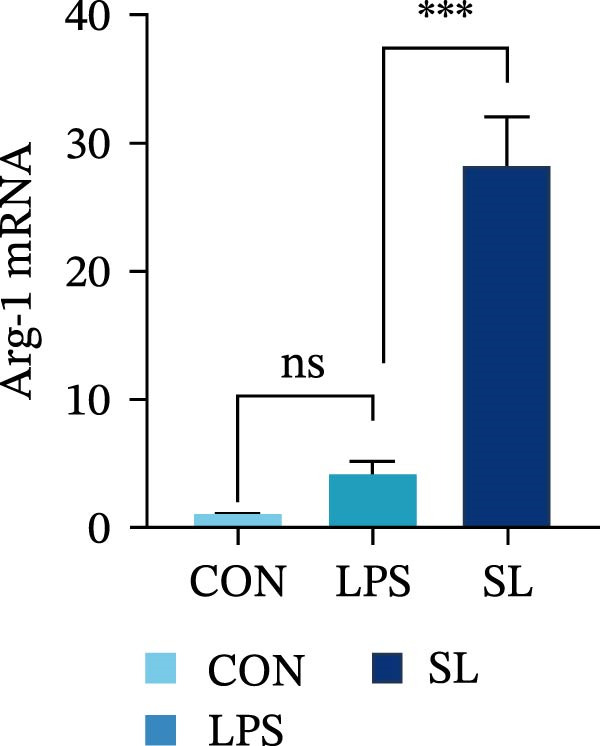
(G)

**Figure:**
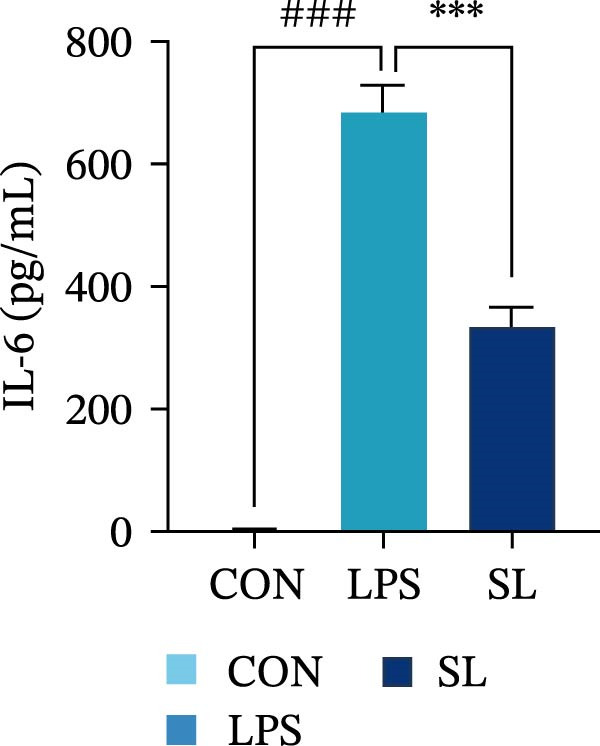
(H)

**Figure:**
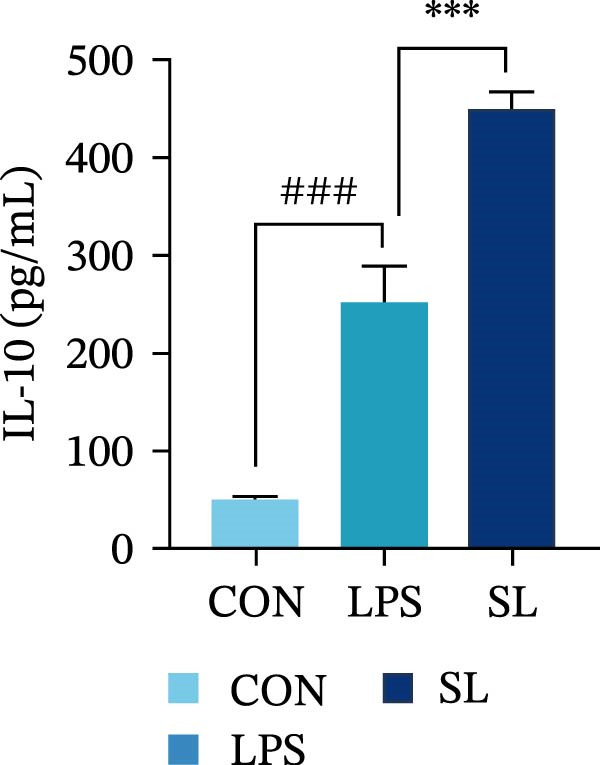
(I)

### 3.6. The Inhibition of Renal TEC Pyroptosis Caused by Macrophage M1 Polarization Was Achieved by the Administration of SL Decoction

RAW264.7 and TCMK‐1 cells were co‐cultured under HG conditions (Figure 6A). Prior to co‐culture, RAW264.7 macrophages were pre‐treated with LPS (1 μM) and SL decoction‐containing serum for 24 h. Following this, the RAW264.7 macrophages were co‐cultured with renal tubular cells, with the aim of removing the effect of LPS and SL decoction‐containing serum on renal tubular cells. Subsequently, the levels of TLR4, NF‐κB p65, GSDMD, Caspase‐1, and NLRP3 were detected by qRT‐PCR. The levels of TLR4, NF‐κB p65, GSDMD, Caspase‐1, and NLRP3 in renal tubular cells were markedly elevated following co‐culture with macrophages in HG (Figure 6B–F). Following administration via SL decoction, there was a notable reduction in the expression levels of TLR4, NF‐κB, GSDMD, and Caspase‐1, accompanied by a slight, though not statistically significant, decline in NLRP3 (Figure 6B–F). The collective findings indicate that the TLR4 pathway is a key mediator of tubular injury in macrophages and DN.

Figure 6RAW264.7 cells were seeded in the top compartment of Transwell, separated by a porous membrane from TCMK‐1 cells culture medium (A). The mRNA level of TLR4, NF‐κB p65, Caspase‐1, NLRP3, and GSDMD (*n* = 3) (B–F).

**Figure:**
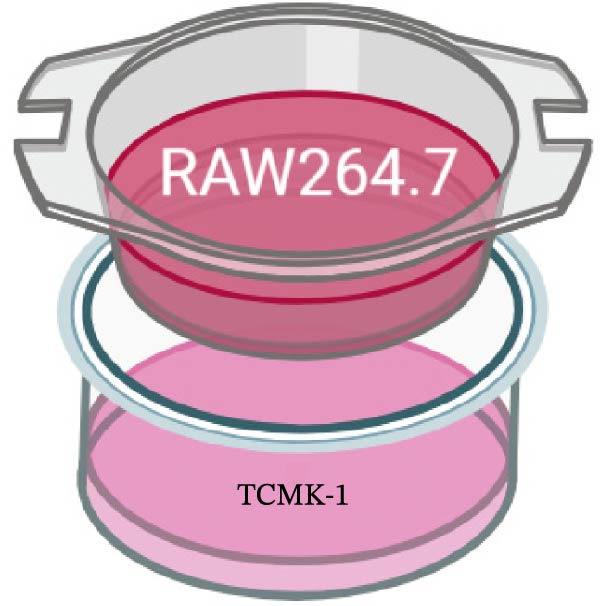
(A)

**Figure:**
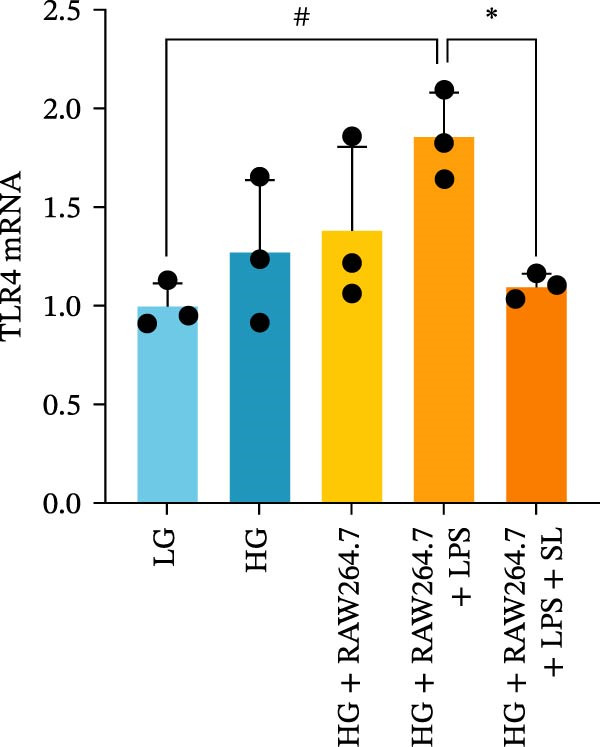
(B)

**Figure:**
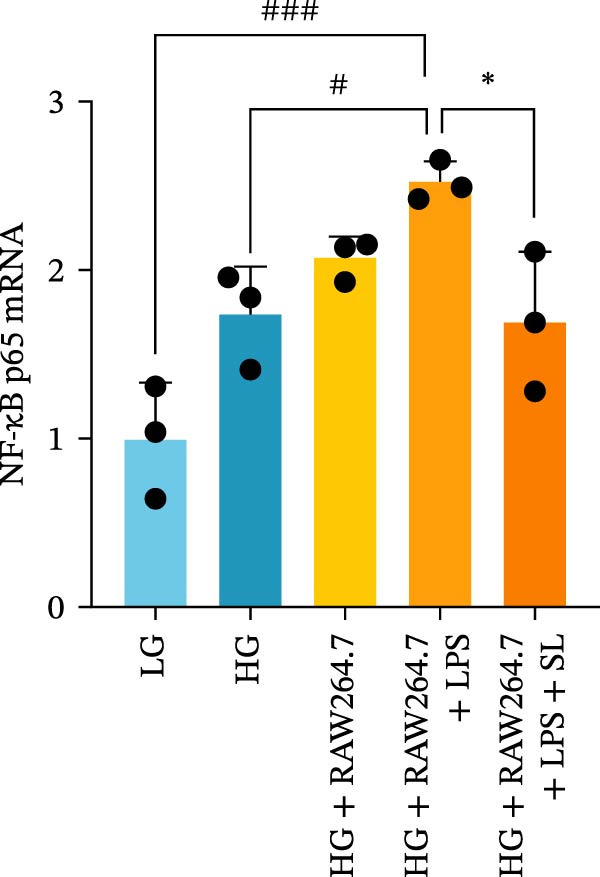
(C)

**Figure:**
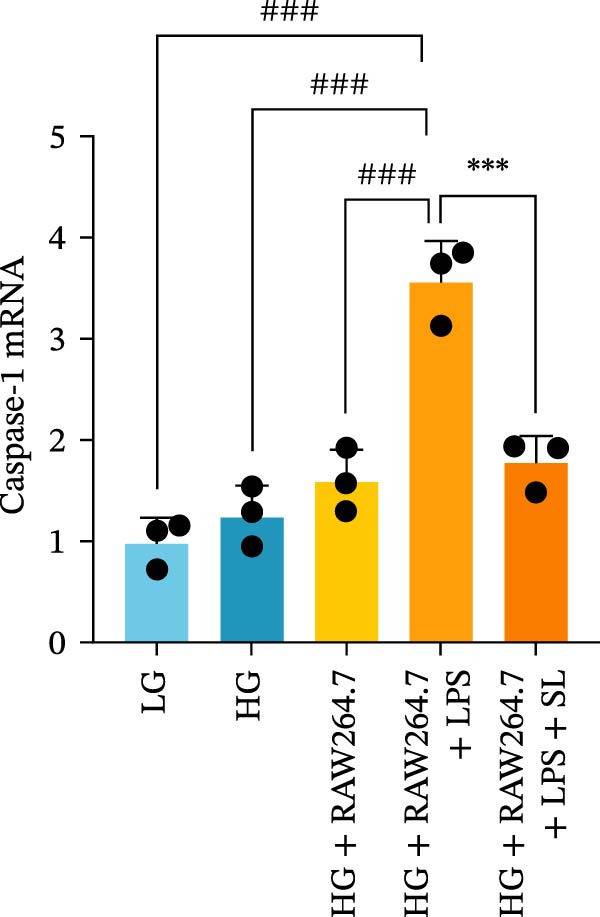
(D)

**Figure:**
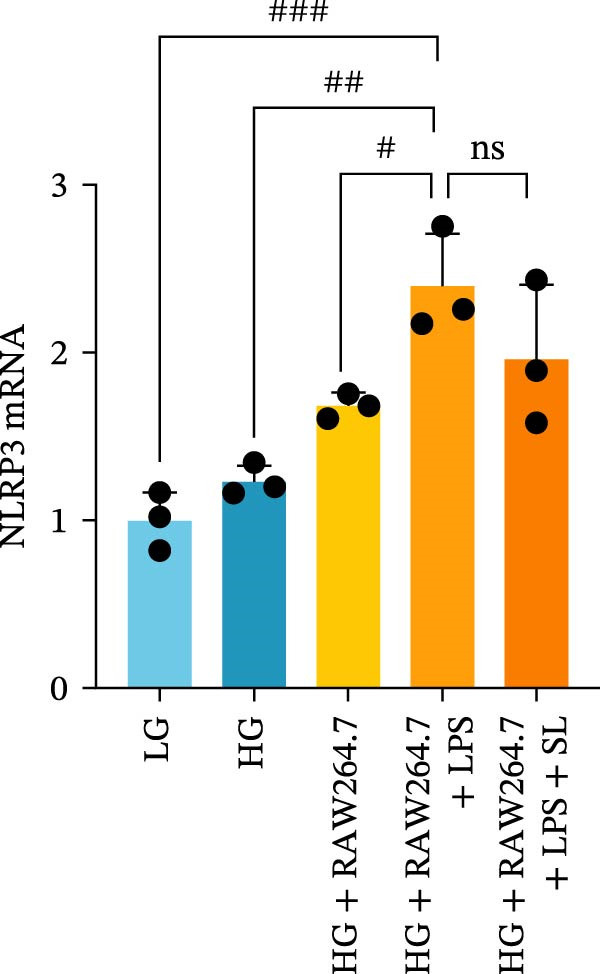
(E)

**Figure:**
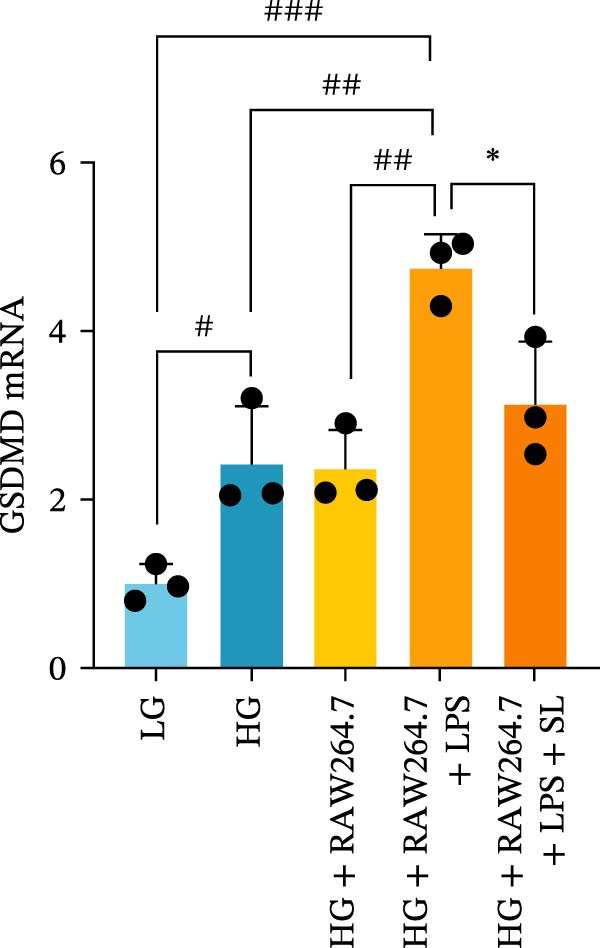
(F)

## 4. Discussion

DN represents a significant complication of diabetes mellitus, exerting a profound impact on the microvascular system and serving as a primary contributor to the global prevalence of CKD. Statistical data indicates that ~30%–40% of individuals with diabetes will develop DN, with 5%–10% of these cases ultimately progressing to ESRD [1]. The objective of this study was to investigate the role of SL decoction in the treatment of DN. The findings indicated that SL decoction impeded the polarization of macrophages M1 in DN, thereby curbing the activation of the TLR4 signaling pathway and renal TEC pyroptosis. The findings of this study provide a basis for the potential clinical application of SL decoction in the management of DN.

In this study, db/db mice were employed as an animal model of DN. db/db mice exhibit a genetic defect in the leptin receptor, which can result in the development of obesity and insulin resistance. They are now widely utilized in the investigation of type 2 diabetes mellitus [16]. Additionally, db/db mice have been observed to exhibit augmented renal macrophage infiltration in comparison to db/m mice [17]. Metformin was selected as the positive control drug in this study due to its nephroprotective effects and its capacity to modulate the mechanism of TLR4/NF‐κB in the management of DN [18, 19]. In this study, fasting blood glucose, blood creatinine, urea nitrogen, and ACR were examined in db/db mice. The results demonstrated that blood glucose, urea nitrogen, and ACR were significantly elevated in the DN group of mice in comparison to the blank control group. Concurrently, renal pathology exhibited pathological manifestations, including glomerular hypertrophy, proliferation of mesangial cells, expansion of the mesangial matrix, narrowing of the renal capsule, blurring of the luminal rim of the renal tubule, and renal interstitial haematochezia and edema. Additionally, there was an increase in inflammatory cell infiltration compared to the control group, which was consistent with the pathological features of the kidneys of DN. SL decoction has been demonstrated to reduce blood creatinine and urea nitrogen in db/db mice. Furthermore, it has been observed to alleviate renal pathological manifestations, including glomerular hypertrophy, proliferation of tethered membrane cells, and blurring of tubular luminal rims. Additionally, the administration of this combination has been shown to attenuate inflammatory cell infiltration. These findings collectively suggest that the SL decoction has the potential to protect renal function in DN.

It is becoming increasingly clear that renal inflammation plays an important role in the pathogenesis of DN. This is evidenced by the activation of inflammatory signaling pathways, the release of inflammatory cytokines and the infiltration of immune cells. It has been demonstrated that macrophages represent the most prevalent infiltrating leukocyte in the kidneys of patients with DN, and are associated with a reduction in renal function in DN. Macrophages are primarily distinguished by two main phenotypes, designated as M1 and M2 [20]. M1‐type macrophages have been linked to pro‐inflammatory effects and can be stimulated by pathogen‐associated molecular patterns (PAMPs), danger‐associated molecular patterns (DAMPs), interferon‐gamma (IFN‐γ), lipopolysaccharide (LPS), and pro‐inflammatory cytokines, resulting in the secretion of inflammatory factors such as IL‐6, IL‐1β, and TNF‐α. These factors contribute to the exacerbation of renal inflammation and injury to renal parenchymal cells [7]. The results of clinical studies indicate that the number of glomerular and tubulointerstitial macrophages is elevated in patients diagnosed with DN relative to patients with DN but without renal abnormalities and diabetic patients without renal complications [21]. In the initial stages of DN (nephropathological stages I and IIa), resident and recruited macrophages undergo differentiation into pro‐inflammatory M1 macrophages in significant numbers [22]. Conversely, in DN, the number of M2 macrophages is further increased in the late stages (nephropathological stage III) [23]. Macrophages and their phenotypes can be labeled by specific markers. The identification of macrophages can be achieved through the use of markers such as F4/80 and CD68, which are surface molecular markers. Additionally, the marker iNOS is commonly used for the identification of M1‐type macrophages and is now widely employed in this context. The TLR4 signaling pathway has been demonstrated to induce macrophage M1 polarization. Activation of TLR4 results in phosphorylation of the IKK2 complex, which in turn phosphorylates IκBα. This releases NF‐κB p65, which rapidly translocates to the nucleus, where it is phosphorylated to p‐p65. This process induces macrophage M1 polarization, including the high expression of iNOS and the subsequent release of inflammatory factors such as IL‐6, IL‐1β, and TNF‐α [24]. Research indicates that traditional Chinese medicine may inhibit the progression of DN by regulating macrophages [25]. The findings of the present study demonstrated that SL decoction markedly diminished the expression of the macrophage marker F4/80 and the co‐localization of CD68/iNOS in the kidneys of db/db mice, and reduced serum levels of IL‐6, IL‐1β, and TNF‐α. In cellular experiments, SL decoction was observed to inhibit LPS‐induced high TLR4 expression and nuclear translocation of NF‐κB p65, while also upregulating Arg‐1 and downregulating iNOS. Furthermore, ELISA of cell supernatants demonstrated that SL decoction diminished the secretion of the pro‐inflammatory factor IL‐6 while enhancing the secretion of the anti‐inflammatory factor IL‐10. The results of these studies indicate that SL decoction inhibits the infiltration of DN renal M1‐type macrophages and reduces the release of inflammatory factors. This appears to be due to a mechanism involving the inhibition of TLR4/NF‐κB activation.

Recent studies have demonstrated that renal TEC pyroptosis is a pivotal mechanism in the pathogenesis of DN. The injury of renal TECs represents a pivotal mechanism underlying the development of microalbuminuria in DN [26]. The pathogenesis of DN is associated with macrophage‐induced renal TEC injury [27]. A high glucose environment has been demonstrated to induce NF‐κB activation and M1 polarization in macrophages, which in turn has been shown to lead to renal inflammation, fibrosis and necrotic apoptosis in RTECs [28]. Recent studies have demonstrated that M1‐type macrophages can induce cellular pyroptosis by activating NLRP3 inflammatory vesicles in TECs [29]. Pyroptosis is a caspase‐1‐dependent cell death pathway, which is characterized by a number of distinctive features, including cell swelling, formation of cell membrane pores, rupture of the cell membrane, cleavage of DNA, activation of inflammatory vesicles, release of cellular contents and inflammatory mediators. It has the property of promoting inflammatory responses [2]. The activation of inflammatory vesicles represents a pivotal event in the induction of cellular pyroptosis, with the NLRP3 inflammatory vesicle serving as a primary exemplar. In response to microbial infection and cellular damage, activated caspase‐1 cleaves precursors of cytokines IL‐1β and IL‐18 [3]. Caspase‐1 also activates GSDMD, which results in the formation of membrane pores in the plasma membrane, the mediation of the secretion of IL‐1β and IL‐18, and the induction of cellular pyroptosis. TLR4 is highly expressed in the renal tubular mesenchyme [30], and the TLR4/NF‐κB pathway is one of the upstream pathways that induces cellular pyroptosis. This pathway has been demonstrated to be involved in renal inflammation in DN [31]. The present study demonstrated that renal TLR4, NF‐κB p65, and p‐NF‐κB p65 were decreased in db/db mice following SL decoction intervention, as evidenced by IHC and WB. Additionally, the expression of pyroptosis‐related proteins, including NLRP3, Caspase‐1, GSDMD, GSDMD‐NT, IL‐18, and IL‐1β, was also reduced, suggesting that SL decoction inhibits cellular pyroptosis in the development of DN. Furthermore, macrophage RAW264.7 and renal TEC TCMK‐1 were co‐cultured using Transwell to observe the impact of macrophage M1 polarization on renal TEC pyroptosis. The results demonstrated that following co‐culture with SL decoction‐intervened RAW264.7 macrophages, renal TEC TLR4, and NF‐κB p65 mRNA exhibited a notable decline in comparison to the preceding levels. Similarly, GSDMD, caspase‐1, NLRP3 mRNA, and GSDMD and GSDMD‐NT protein levels exhibited a marked reduction. It has been proposed that M1‐polarized macrophages may be responsible for the induction of pyroptosis in co‐cultured renal TECs. Furthermore, it has been suggested that SL decoction intervention may prove an effective means of inhibiting this process.

Nevertheless, the present study was limited to co‐culture experiments on macrophages and renal TECs, with the aim of preliminarily investigating the role of SL decoction in inhibiting macrophage M1 polarization to attenuate renal TEC pyroptosis. However, its specific mechanism remains unclear. Further studies are planned to elucidate the mechanisms of action of macrophage‐secreted proteins or exosomes on renal TECs.

## 5. Conclusion

This study employed network pharmacology and in vivo and in vitro experiments to confirm that SL decoction can improve renal function in patients with DN. The results demonstrated that the mechanism of action of SL decoction is related to regulating the M1 polarization of macrophages in the DN kidney and inhibiting the pyroptosis of renal TECs.

**Nomenclature**
6hUTP: 6 h urine total protein quantity
ACR: albumin/urine creatinine ratio
CKD: chronic kidney disease
DAMPs: danger‐associated molecular patterns
DN: Diabetic nephropathy
ESRD: end‐stage kidney disease
H–E: hematoxylin–eosin
IF: Immunofluorescence
IFN‐γ: interferon‐gamma
IHC: Immunohistochemistry
LPS: lipopolysaccharide
PAMPs: pathogen‐associated molecular patterns
PAS: periodate‐Schiff staining
RT‐qPCR: RNA extraction and real‐time quantitative polymerase chain reaction
SL: Shenlian
TEC: tubular epithelial cell.

## Author Contributions

**Xiaozhe Fu:** methodology, investigation, data curation, writing – original draft. **Qiang Fu:** conceptualization, investigation, formal analysis, visualization, resources, writing – original draft, writing – review & editing. **Yuanyuan Liu:** investigation. **Yifan Liu:** investigation. **Junheng Wang:** investigation. **Jie Hu:** investigation. **Chuxiao Tian:** investigation. **Wenqian Yu:** investigation. **Yixuan Ren:** investigation. **Weijun Huang:** investigation. **Jinxi Zhao:** conceptualization, funding acquisition, supervision, project administration, writing – review & editing.

## Funding

This study was supported by National Natural Science Foundation of China, 82004302 82074354, the Sixth Batch of National Excellent Talent Training Program for Traditional Chinese Medicine Clinicians.

## Conflicts of Interest

The authors declare no conflicts of interest.

## Supporting Information

Additional supporting information can be found online in the Supporting Information section.

## Supporting information


**Supporting Information 1** Table S1: Potential targets for active ingredients in SL decoction.

**Supporting Information 2** Table S2: Genes associated with diabetic nephropathy.

**Supporting Information 3** Table S3: GO enrichment analysis.

**Supporting Information 4** Table S4: KEGG enrichment analysis.

## Data Availability

The data that support the findings of this study are available from the corresponding author upon reasonable request.
